# The associations between social support, self-regulatory fatigue, and health-promoting behaviors among people with type 2 diabetes mellitus: a cross-sectional survey

**DOI:** 10.3389/fpubh.2023.1281065

**Published:** 2023-12-14

**Authors:** Xin Wang, Fan Zhang, Yuanhui Ge, Yiqian Ding, Tao Liu

**Affiliations:** ^1^Department of Nursing, Jinzhou Medical University, Jinzhou, China; ^2^Nursing Department of Huaian Hospital of Huaian City, Huaian, China

**Keywords:** self-regulatory fatigue, social support, health-promoting behaviors, type 2 diabetes mellitus, mediating effect

## Abstract

**Background:**

The prevalence of diabetes in China is increasing annually, posing a serious public health challenge. Health-promoting behaviors are crucial for enhancing the quality of life in individuals with type 2 diabetes. However, the relationship between self-regulatory fatigue in type 2 diabetes, social support, and health-promoting behaviors remains unclear.

**Objective:**

This study aimed to explore the interconnections among social support, self-regulatory fatigue, and health-promoting behaviors, as well as to examine the mediating role of self-regulatory fatigue in patients with type 2 diabetes mellitus.

**Methods:**

A cross-sectional design was employed using the Self-Regulatory Fatigue Scale (SRF-S), the Social Support Rating Scale (SSRS), and the Health Promotion Scale for People with Diabetes Mellitus (T2DHPS). These scales assessed social support, self-regulatory fatigue, and health-promoting behaviors in 316 patients with type 2 diabetes mellitus, exploring the relationships among these variables. SPSS and AMOS were used for statistical analysis to investigate the mediating effects.

**Results:**

Social support in type 2 diabetes mellitus positively predicted health-promoting behaviors (β = 0.401, *p* < 0.001). The regression coefficients of self-regulatory fatigue in patients with type 2 diabetes mellitus social support (β = −0.502, *p* < 0.001), and health-promoting behaviors (β = −0.331, *p* < 0.001), both exhibiting significant differences. Self-regulatory fatigue mediated the relationship between social support and health-promoting behaviors in these patients, with a mediation effect of 0.166, consisting of 29.28% of the total effect.

**Conclusion:**

A significant interplay exists among social support, self-regulatory fatigue, and health-promoting behaviors in patients with type 2 diabetes mellitus. The findings suggest that self-regulatory fatigue mediates the relationship between social support and health-promoting behaviors. Healthcare professionals should focus on enhancing patients' social support to mitigate self-regulatory fatigue and improve health behaviors.

## 1 Introduction

Diabetes mellitus is a major public health concern in China, ranking third among chronic non-communicable diseases, after cardiovascular and cerebrovascular disorders and cancer, in posing a significant health threat (1, 2). The International Diabetes Federation reports that the global diabetes population reached 537 million in 2021, with projections suggesting an increase to 783 million by 2045 (3). In China, the prevalence rate of diabetes is 11.2% (4), the highest globally, with type 2 diabetes mellitus (T2DM) constituting approximately 90% of cases (5). Notably, there is a geographic variation in the prevalence of diabetes across China (6), with higher incidence rates in the northern regions, possibly due to dietary factors (7). T2DM, one of the world's fastest-growing diseases, is expected to continue straining healthcare systems and causing significant personal and economic burdens (3).

Social support, which includes the aid patients perceive and receive from their social networks, such as interactions with friends, family, neighbors, and coworkers, plays a vital role in reducing psychological stress and enhancing social adaptation (8). Other important factors influencing health-promoting behaviors include psychological resilience, literacy, and interpersonal interactions (9, 10). Cultural differences are significant in diabetes management (11), prompting the Chinese Health Commission to provide tailored food and nutritional guidelines for T2DM patients based on regional dietary and cultural variations (12). T2DM patients often need to adhere to strict, long-term health behaviors, such as consistent exercise, calorie restriction, weight management, and blood glucose monitoring (13, 14). Studies have shown that social support is a critical social determinant for the self-management of diabetes mellitus patients, and is far from sufficient if carried out by patients individually (15, 16). Past research has demonstrated that diabetic patients benefitted from strong social support in both their physical and emotional health (17). However, the level of social support for T2DM patients in China needs improvement (18). Xu et al. indicate that T2DM patients receive a medium degree of social support (18), suggesting the necessity to enhance social support for their mental and physical well-being.

In 1987, Pender introduced the concept of health-promoting behaviors, stating that actions individuals undertake to maintain a positive state across all life aspects (19). The health-promoting behavior model (20) highlights the significant influence of interpersonal ties on such behaviors, with strong interpersonal connections being crucial for acquiring social support. This model suggests that individual traits and experiences can impact health behaviors either directly, through previous behavioral habits, or indirectly, by affecting specific behaviors, mental cognitions, and emotions. Health-promoting behaviors are beneficial for physical and emotional well-being, aiding in disease prevention and health maintenance or improvement (21). However, Xu et al. noted that 80% of T2DM patients have inadequate blood glucose control and related complications (22), resulting in a low level of health promotion and a diminished sense of self-care. Hence, it is imperative for T2DM patients to not only receive therapeutic treatment but also enhance their risk factor management and health-promoting behaviors. Furthermore, the link between social support and health-promoting behaviors has been established in various populations (23, 24), but the specific mechanisms of their roles in the diabetic population remain underexplored.

T2DM necessitates long-term management involving dietary monitoring, oral hypoglycemic medications, and insulin injections, due to the disease's prolonged nature, recurrence risk, comorbidities, complex disease progression, and the challenge of achieving a complete cure. This continuous demand often leads to self-regulatory fatigue in patients (25). Self-regulatory fatigue is defined as a temporary decrease in an individual's capacity or willingness to engage in self-directed activities where self-control is required (26). It manifests as a diminished ability to regulate emotions, thoughts, and behaviors. According to the energy model of self-control, an individual's self-control capacity is finite (26), with the performance of an initial task affecting subsequent tasks in the control cycle, a phenomenon known as ego depletion. Nes et al. (27) described self-regulatory fatigue as a persistent fatigue state caused by long-term resource depletion due to factors like economic stress, anxiety, and depression, and it is more challenging to recover from compared to ego depletion, which is transient. Self-regulatory fatigue impacts individuals with chronic illnesses more significantly and for a longer duration than healthy individuals (28). T2DM patients, being at risk of developing self-regulatory fatigue, often experience ongoing fatigue when engaging in self-controlled activities. Hence, this study hypothesizes that self-regulatory fatigue may act as a mediator between social support and health-promoting behaviors. Moreover, most research on self-regulatory fatigue in China has focused on individuals with chronic pain (29), with limited exploration into self-regulatory fatigue in T2DM.

In summary, this study aims to examine the interplay between self-regulatory fatigue, social support, and health-promoting behaviors in T2DM patients. It specifically focuses on the potential mediating role of self-regulatory fatigue in the relationship between social support and health-promoting behaviors. This investigation intends to contribute to the existing body of knowledge on self-regulatory fatigue, particularly in the context of T2DM. An essential aspect of this research is to determine if the self-regulation of fatigue status serves as a predictor for higher levels of health-promoting behaviors in these patients. The findings are expected to enable medical staff to implement targeted strategies, considering social support and self-regulatory fatigue. These insights aim to provide new directions for managing patient care during clinical interactions or follow-ups and to establish a new benchmark for enhancing health-promoting behaviors among T2DM patients.

## 2 Objects and methods

### 2.1 Participants and procedures

This study employed a cross-sectional survey design. Participants were type 2 diabetes mellitus (T2DM) patients recruited from the endocrinology department of a tertiary hospital in Huai'an City, Jiangsu Province, China, from May to August 2023 through convenient sampling. The inclusion criteria were as follows: Age above 18; Diagnosis of T2DM according to the World Health Organization's 1999 criteria (5); Disease duration exceeding 3 months; Basic language comprehension and response abilities; Awareness of the diagnosis and consent to participate in the study. Exclusion criteria included: Mental and cognitive impairments and multiple serious illnesses.

### 2.2 Research tools

#### 2.2.1 General information questionnaire

The questionnaire, designed by researchers, gathered data on gender, age, education level, occupation, place of residence, marital status, monthly household income per capita, years living with diabetes, presence of diabetic complications, and other chronic conditions.

#### 2.2.2 Social support rating scale

Developed by Xiao (30), the Social Support Rating Scale (SSRS) is widely used in China to assess levels of social support. The scale encompasses 10 items across three dimensions: 3 items for objective support, 4 items for subjective support, and 3 items for support utilization. Items 1–4 and 8–10 are assessed using a 4-point Likert scale. Question 5 contains 5 options (A to E), each scored from 1 to 4 points, reflecting a range from “no” to “full support”. For questions 6 and 7, answers are scored 1–4 points based on the identified sources of support, while “no source” responses receive 0 points. The total score correlates with the level of social support, categorized as: low (≤22), moderate (23–44), or high (≥45). In this study, the SSRS demonstrated robust reliability and validity, with a Cronbach's alpha of 0.806, a Kaiser-Meyer-Olkin (KMO) measure of 0.848, and an approximate chi-square of 1,415.524 (*p* < 0.001) in Bartlett's test of sphericity.

#### 2.2.3 Self-Regulatory Fatigue Scale

The Self-Regulatory Fatigue Scale (SRF-S), initially developed by Nes et al. in 2013, was translated and adapted into Chinese by Wang et al. in 2015 (27, 31). Research has shown that this scale is suitable for Chinese residents and possesses good reliability. The SRF-S comprises 16 items, divided into three dimensions: cognitive control (6 items), emotional control (5 items), and behavioral control (6 items). Responses are scored using a 5-point Likert scale (strongly oppose = 1, disapprove = 2, uncertai*n* = 3, approve = 4, strongly agree = 5), with the total score ranging from 16 to 80. Higher scores indicate increased levels of self-regulatory fatigue. In this study, the SRF-S demonstrated excellent reliability and validity, with a Cronbach's alpha of 0.884, a KMO measure of 0.904, and an approximate chi-square of 2019.408 (*p* < 0.001) in Bartlett's test of sphericity.

#### 2.2.4 The health promotion scale for people with diabetes

Developed by Chen et al. in 2013, the T2DHPS assesses health-promoting behaviors in T2DM patients (32). Cao et al. evaluated the scale's reliability and validity, confirming its applicability to Chinese residents (33). The T2DHPS consists of 28 items across six dimensions: physical activity (7 items), risk reduction (7 items), stress management (5 items), enjoying life (3 items), health responsibility (3 items), and a healthy diet (3 items). The scale uses a 5-point Likert scale (neve*r* = 1, occasionally = 2, about half = 3, often = 4, always = 5), with the total score ranging from 28 to 140. Higher scores indicate greater levels of health promotion in diabetic patients. In this study, the T2DHPS showed high reliability and validity, with a Cronbach's alpha of 0.884, a KMO measure of 0.904, and an approximate chi-square of 2019.408 (*p* < 0.001) for Bartlett's test of sphericity.

### 2.3 Ethical approval and data collection

The study received ethical approval from the Jinzhou Medical University Ethics Committee (JZMULL2023067). Participants were informed by the research team members about the study's purpose and significance. All participants provided informed consent and voluntarily took part in the study. Trained team members distributed and collected the questionnaires in a one-on-one manner in the wards. To ensure confidentiality, all questionnaires were completed anonymously, and participants were assisted with any omissions during the on-site collection. For individuals with limited literacy, researchers read the questionnaires aloud and recorded the responses. The study's questionnaire comprised 22 variables. According to Kendall's rough estimation method for sample size (34), the sample should be 5–10 times the number of variables, suggesting a range of 110–220 cases. Accounting for a 20% sample attrition rate and the potential error due to convenience sampling, the estimated sample size was adjusted to 132–264 cases. Considering structural equation modeling, a sample size of 200 or more was preferred (35). To ensure result reliability, 350 questionnaires were distributed, resulting in 316 valid responses and a validity rate of 90.28%.

### 2.4 Statistical methods

The data collected in this study were analyzed using two statistical software tools: SPSS 27.0 and AMOS 24.0. These tools are commonly utilized in medical research for their user-friendly interface and capability to perform a wide range of statistical functions without requiring complex programming (36). To evaluate the relationships between social support, self-regulatory fatigue, and health-promoting behaviors, descriptive statistics were employed, including mean, standard deviation, skewness, and kurtosis, using Pearson's correlation analysis under a normal distribution. Harman's single factor test was applied to assess potential common methodological biases (37). A result exceeding 40% in this test indicates a significant discrepancy between the measured outcomes and actual conditions.

Structural equation modeling (AMOS) was utilized for path analysis. The normality of the distribution was first assessed by calculating skewness and kurtosis. In this study, skewness values ranged between −0.50 and 0.48, and kurtosis values were between −0.97 and 1.12. These values, falling within the ± 2 range, are generally considered acceptable for assuming a normal distribution (38). This finding indicates the data's suitability for AMOS analysis. To determine the goodness of fit of the structural model, various indices were used, including the Comparative Fit Index (CFI), Goodness of Fit Index (GFI), Incremental Fit Index (IFI), Root Mean Square Error of Approximation (RMSEA), and the chi-square to degrees of freedom ratio (χ^2^/df). The significance of the direct and indirect effects of the variables within the AMOS framework was ascertained using a 95% confidence interval.

## 3 Results

### 3.1 Characteristics of participants

The study included 316 participants, with a nearly equal distribution of genders: 49.7% men and 50.3% women. Detailed demographic characteristics are provided in Table 1.

**Table 1:** The demographics and medical characteristics of participants (*n* = 316).

**Factors**	**Group**	***n***	**%**
Gender	Male	157	49.7
	Female	159	50.3
Age (years)	18–44	57	18.0
	45–59	102	32.3
	≥60	157	49.7
Education	Elementary school and lower	97	30.7
	Junior middle school	70	22.2
	Secondary technical school or Senior high school	69	21.8
	Junior college	36	11.4
	Bachelor's degree and above	44	13.9
Occupation	Farmer	70	22.2
	Worker	38	12.0
	Staff	47	14.9
	Freelancer	47	14.9
	Resignation/retirement	59	18.7
	Unemployed	55	17.4
Residence	Rural	131	41.5
	City	185	58.5
Marital status	Unmarried	22	7.0
	Married	210	66.5
	Divorce	47	14.9
	Widowed	37	11.7
Per-capita family income	< 1,000¥	17	5.4
	1,000–1,999¥	51	16.1
	2,000–2,999¥	89	28.2
	3,000–3,999¥	87	27.5
	≥4,000¥	72	22.8
Number of years of illness	0–10	176	55.7
	>10	140	44.3
Complications	No	136	43.0
	Yes	180	57.0
Other chronic illnesses	No	172	43.7
	Hypertension	130	33.0
	Hyperlipidemia	43	10.9
	Coronary heart disease	32	8.1
	Others	17	4.3

### 3.2 Scores of social support, self-regulatory fatigue, and health-promoting behaviors of T2DM

The overall social support score of T2DM was 33.13 ± 9.62 (skewness = 0.18; kurtosis = −0.24), the overall self-regulatory fatigue score was 47.95 ± 8.30 (skewness = −0.19; kurtosis = −0.23), and the overall health-promoting behavior score was 71.57 ± 13.28 (skewness = 0.26; kurtosis = 0.47). See Table 2 for details.

**Table 2:** Scores of social support, self-regulatory fatigue and health-promoting behaviors of T2DM (*n* = 316).

**Variable**	**Min**	**Max**	**Mean ±SD**	**Skewness**	**Kurtosis**
**Total score of social support**	**12.00**	**61.00**	**33.13** **±9.62**	**0.18**	**−0.24**
Objective support	1.00	20.00	8.19 ± 3.55	0.05	−0.06
Subjective support	8.00	31.00	18.36 ± 5.10	0.31	−0.39
Utilization of support	3.00	12.00	6.58 ± 2.60	0.33	−0.97
**Total score of self-regulatory fatigue**	**23.00**	**72.00**	**47.95** **±8.30**	**−0.19**	**−0.23**
Cognitive control	8.00	28.00	18.95 ± 3.86	−0.50	−0.25
Behavior control	6.00	24.00	14.69 ± 3.29	−0.14	−0.33
Emotion control	6.00	25.00	14.31 ± 3.28	0.10	−0.51
**Total score of health-promoting behaviors**	**35.00**	**126.00**	**71.57** **±13.28**	**0.26**	**0.47**
Physical activity	7.00	34.00	16.09 ± 5.08	0.48	0.18
Risk reduction	8.00	35.00	18.67 ± 4.76	0.42	0.40
Stress management	5.00	24.00	13.63 ± 2.95	0.32	1.12
Enjoy life	3.00	14.00	7.83 ± 2.28	0.12	−0.46
Health responsibility	3.00	15.00	7.85 ± 2.08	0.07	0.04
Healthy diet	3.00	14.00	7.51 ± 2.13	0.35	0.04

### 3.3 Relationship between social support, self-regulatory fatigue, and health-promoting behaviors in T2DM

Social support was negatively correlated with self-regulatory fatigue (*r* = −0.431, *p* < 0.001), social support was positively correlated with health-promoting behavior (*r* = 0.424, *p* < 0.001), self-regulatory fatigue was negatively correlated with health-promoting behavior (*r* = −0.408, *p* < 0.001). The results are shown in Table 3.

**Table 3:** Correlation analysis results of social support, self-regulatory fatigue and health-promoting behaviors of T2DM (*n* = 316).

**Variable**	**Social support**	**Self-regulatory fatigue**	**Health-promoting behaviors**
Social support	1		
Self-regulatory fatigue	−0.431^**^	1	
Health-promoting behaviors	0.424^**^	−0.408^**^	1

** Significant correlation at 0.01 level (bilateral).

### 3.4 Common method deviation inspection

Harman's single-factor test was conducted to assess common method bias. The variance explained by the first factor was 17.938%, well below the 40% threshold, indicating no significant common method variance. Additionally, 25 components had eigenvalues greater than 1. As a result, this study does not exhibit any significant common methodological variations.

### 3.5 The mediating role of self-regulatory fatigue in the relationship between social support and health-promoting behaviors in T2DM

**Figure 1:**
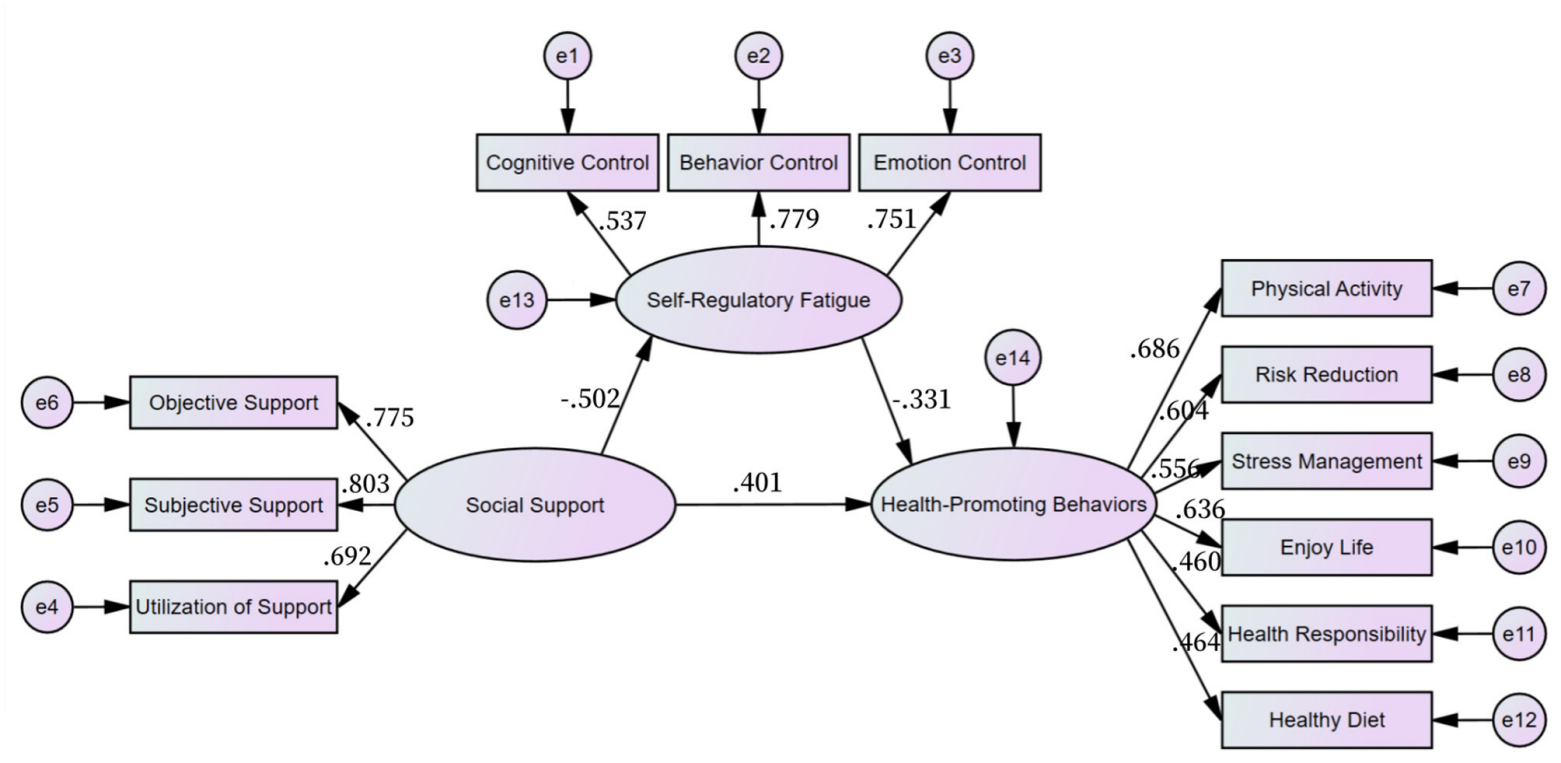
A mediated model of social support, self-regulatory fatigue and health-promoting behaviors of T2DM.

The study utilized an Amos bias-corrected nonparametric percentile bootstrap approach to confirm the significance of the mediating effect. To minimize Type I errors impacting statistical inference, a random sample of 2000 was selected from the original sample size of 316. The structural equation model included social support (comprising 3 latent variables) as the predictor, self-regulatory fatigue (3 latent variables) as the mediator, and health-promoting behaviors (6 latent variables) as the outcome, illustrated in Figure 1.

Each fitting index of the structural equation model: CFI = 0.918, GFI = 0.932, IFI = 0.919, RMSEA = 0.073, χ^2^/df = 2.688, all of which were in line with the ideal criteria, good model adaptability. These results are detailed in Table 4.

**Table 4:** Structural equation model fitting index (*n* = 316).

**Project**	**χ^2^**	**χ^2^/df**	**RMSEA**	**GFI**	**CFI**	**IFI**
Fitting index	137.093	2.688	0.073	0.932	0.918	0.919
Acceptable standards	-	< 3	< 0.08	>0.9	>0.9	>0.9

**χ^2^**/df Maximum likelihood ratio χ2 values/degrees of freedom, GFI Goodness of fit index, CFI Comparative fit index, IFI Value added index, RMSEA Mean square sum square root of progressive residuals.

### 3.6 Estimation of parameters related to social support, self-regulatory fatigue, and health-promoting behaviors in T2DM

The regression analysis indicated that social support in T2DM positively predicted health-promoting behaviors (β = 0.401, *p* < 0.001). Additionally, the regression coefficients of self-regulatory fatigue significantly influenced both the paths from T2DM social support (β = −0.502, *p* < 0.001) and health-promoting behaviors (β = −0.331, *p* < 0.001). These results are presented in Table 5 and Figure 1.

**Table 5:** Estimated parameters and 95% CI between social support, self-regulatory fatigue and health-promoting behaviors of T2DM (*n* = 316).

**Project**	**Unstandardized coefficients**	**Standardized coefficients**	**SE**	**Z(sig.)**	**confidence interval**	
					**Boot CI Upper**	**Boot CI Lower**
Social support → self-regulatory fatigue	−0.289	−0.502	0.051	*p* < 0.001	−0.424	−0.166
Self-regulatory fatigue → health-promoting behaviors	−0.476	−0.331	0.127	*p* < 0.001	−0.805	−0.184
Social support → health-promoting behaviors	0.333	0.401	0.070	*p* < 0.001	0.152	0.531

The analysis revealed that self-regulatory fatigue indirectly predicted the effect of social support on health-promoting behaviors in T2DM, with a standardized path coefficient of (−0.502) ^*^ (−0.331) = 0.166. The total effect of social support on health-promoting behaviors was calculated as (0.166 + 0.401) = 0.567. This finding suggests that self-regulatory fatigue plays a partially mediating role between social support and health-promoting behaviors, accounting for 29.28% of the total effect. Detailed results are shown in Table 6.

**Table 6:** Decomposition table of total effect, direct effect, and mediating effect.

	**Effect value**	**Boot CI upper**	**Boot CI lower**	**Relative effect value**
Total effect	0.567	0.312	0.643	100%
Direct effect	0.401	0.152	0.531	70.70%
Mediating effect	0.166	0.060	0.249	29.30%

The bootstrap method estimates the standard error of indirect effects and the lower and upper limits of 95% confidence intervals.

## 4 Discussion

### 4.1 Levels of social support, self-regulatory fatigue, and health-promoting behaviors in T2DM

The study's findings highlighted that individuals with type 2 diabetes mellitus (T2DM) exhibit moderate levels of self-regulatory fatigue, with an average score of 47.95 ± 8.03 and a scoring rate of 59.94%. This level of self-regulatory fatigue suggests that T2DM patients are experiencing fatigue associated with self-management of their condition. These results align with those from Rod et al.'s study (39). Notably, the cognitive aspect of self-regulatory fatigue received the highest scores, indicating that managing diabetes requires significant mental effort and physical stamina.

In terms of social support, the average score for T2DM patients was 33.13 ± 9.62, classified as moderate according to the scale's criteria. This finding is similar to Yang et al.'s research (40). However, the scores for the utilization of support were relatively low, suggesting that T2DM patients might have difficulties effectively utilizing available social support resources.

The total score for health-promoting behaviors in T2DM patients was 71.57 ± 13.28, with a scoring rate of 51.12%, also indicating a moderate level. This suggests there is room for improvement in T2DM patients' health-promoting behaviors, corroborating the findings of Xu and Baumeister (41, 42). Particularly low scores were observed in the dimensions of exercise and healthy eating. Given that lack of exercise and sedentary lifestyle are high-risk factors for diabetes, and healthy behaviors like maintaining a low-fat diet and regular exercise are essential for its prevention, these areas warrant particular attention (43).

### 4.2 Correlation analysis of social support, self-regulatory fatigue, and health-promoting behaviors of T2DM

This study found a negative correlation between social support and self-regulatory fatigue in T2DM patients, aligning with Zhang et al.'s findings (44). This suggests that higher levels of social support can reduce self-regulatory fatigue in T2DM individuals. Effective management of T2DM involves self-control in diet and regular blood sugar monitoring. When patients perceive strong support from others, they are more inclined to adhere to treatment plans, experience positive emotions, and maintain a constructive attitude toward their illness. They feel valued, supported, and cared for, which reduces self-regulatory fatigue. Conversely, patients with low social support may experience increased depressive symptoms and self-regulatory fatigue, for instance, heightened fear during hypoglycemic episodes. Lack of family and friends' support can hinder positive emotional mobilization, delaying necessary medical interventions.

Furthermore, the study identified a negative correlation between self-regulatory fatigue and health-promoting behaviors. This implies that higher self-regulatory fatigue leads to poorer engagement in health-promoting behaviors. Chronic illness patients, including those with T2DM, often suffer from self-exhaustion (29), which can significantly impair their quality of life and adherence to medical advice. T2DM patients often struggle with depression (45), which can exacerbate their fatigue and reduce active participation in health-promoting behaviors, such as neglecting medical recommendations, skipping blood pressure and glucose checks, adopting unhealthy eating habits, and engaging in minimal physical activity.

Additionally, the study revealed a positive correlation between social support and health-promoting behaviors in T2DM, consistent with Finch et al.'s results (46). Social support, particularly from family and friends is vital in chronic disease management, enhancing self-care in patients (47). Adequate social support can encourage T2DM patients to more diligently follow treatment regimens, access health information more easily (48), improve health-related behaviors, significantly reduce glycated hemoglobin levels (49, 50), and delay the onset of diabetic complications (5, 51). Family members, friends, and romantic partners serve as crucial allies and support managers, bolstering T2DM patients' resolve to engage in health-promoting behaviors.

### 4.3 The mediating role of self-regulatory fatigue

The findings of this study demonstrate the significant mediating role of self-regulatory fatigue in the relationship between social support and health-promoting behaviors in T2DM. The mediation effect was quantified at 0.166, accounting for 29.28% of the total effect. This indicates that social support can influence the level of health-promoting behaviors in T2DM patients through the intermediary of self-regulatory fatigue. Self-regulatory fatigue is a prevalent negative emotion in chronic disease management, intricately linked to cognitive, emotional, and behavioral aspects (52). It can lead to feelings of isolation and reluctance to seek help among patients receiving inadequate social support. Prolonged negative states like anxiety, depression, distress, and loneliness can deplete a patient's limited energy resources (53), significantly impacting their self-control behaviors and leading to a failure in self-regulation, physical exhaustion, and emotional fatigue (54, 55). The consequences of failures in self-regulation can result in reduced self-control and an inability to adopt positive coping styles, adversely affecting the patient's capacity to improve health-promoting behaviors.

Mitigating or avoiding self-regulatory fatigue is possible (56), reducing self-regulatory fatigue through emotional support from others can encourage patients to adopt health-beneficial behaviors. Social support can be categorized into emotional, practical, and behavioral support (57, 58). To help patients feel supported, family members such as partners or children, can accompany them to medical appointments to provide emotional and material support, and assist in understanding diabetes-related biochemical indices, therapeutics, self-care, management and prevention of complications (59). Healthcare professionals should engage more with patients about their conditions, assist in developing personalized diet and exercise plans, and encourage patients to express their support needs (60).

Addressing self-regulatory fatigue involves paying attention to its presence in patients, enhancing thought and behavioral control through goal setting and improved self-efficacy (61), and alleviating the psychological burden it imposes. Maintaining motivation in disease management, changing unhealthy lifestyle habits, and rigorously adhering to health-promoting behaviors such as smoking cessation, alcohol moderation, medication adherence, regular physical exercise, foot care, and blood glucose monitoring are crucial steps in managing diabetes more effectively.

## 5 Strengths and limitations

This study's primary strength lies in its exploration of the relationship between social support, self-regulatory fatigue, and health-promoting behaviors in T2DM, a subject that has not been jointly examined in previous research. The study's identification of moderate levels of self-regulatory fatigue in T2DM is noteworthy, as this aspect has received limited attention in prior studies. The findings underscore the importance of emotional, informational, or tangible support from family, friends, or society in reducing self-regulatory fatigue and promoting physical and psychological adjustments that encourage healthy behaviors in T2DM patients. This research could serve as a foundation for future data collection and cohort studies in broader areas to investigate causal hypotheses.

The study has several limitations inherent to cross-sectional research, including recall bias and challenges in drawing causal conclusions. The sampling population, limited to a tertiary hospital in Jiangsu Province, may affect the generalizability of the findings to the broader T2DM population. Expansion of the sampling scope and level is required for more comprehensive insights. Additionally, the use of a convenience sampling method for the questionnaire may impact the representativeness of the sample.

## 6 Conclusion

This study concludes that T2DM patients experience a state of self-regulatory fatigue and identifies a significant interplay between social support, self-regulatory fatigue, and health-promoting behaviors. Self-regulatory fatigue was found to mediate the relationship between social support and health-promoting behaviors. This finding is crucial for deepening the understanding of the relationship between social support and health-promoting behaviors in T2DM. It suggests that healthcare professionals should facilitate increased social support for T2DM patients through various channels, including wards, outpatient clinics, and community settings. By doing so, they can help reduce self-regulatory fatigue and improve health-promoting behavior levels, thereby enhancing patient health.

## Data availability statement

The raw data supporting the conclusions of this article will be made available by the authors, without undue reservation.

## Ethics statement

The studies involving humans were approved by the Jinzhou Medical University Ethics Committee (JZMULL2023067). The studies were conducted in accordance with the local legislation and institutional requirements. The participants provided their written informed consent to participate in this study.

## Author contributions

XW conducted the research design, statistical analyses, and manuscript writing and revision. FZ, HG, and QD helped with data collection. TL proposed important revisions to the paper. All authors contributed to the article and approved the submitted version.
